# Comprehensive Profiling of Triglycerides in Wild Eastern Mediterranean *Echium* Seed Oil Using Paternò–Büchi Modulated Lipidomics

**DOI:** 10.3390/molecules31030550

**Published:** 2026-02-04

**Authors:** Manal Alhusban, Suha Telfah, Mohammad M. Al-Gharaibeh, Sanaa Bardaweel, Raghad Alkadri, Fang Wei

**Affiliations:** 1 School of Pharmacy, Philadelphia University, Amman 19392, Jordan; malhusban@philadelphia.edu.jo (M.A.);; 2 Department of Pharmaceutical Sciences, Faculty of Pharmacy, Al-Ahliyya Amman University, Amman 19328, Jordan; 3 Department of Plant Production, Faculty of Agriculture, Jordan University of Science and Technology, Irbid 22110, Jordan; mfagharaibeh@just.edu.jo; 4 Department of Pharmaceutical Sciences, School of Pharmacy, The University of Jordan, Amman 11942, Jordan; s.bardaweel@ju.edu.jo; 5 Key Laboratory of Oilseeds Processing of Ministry of Agriculture, Hubei Key Laboratory of Lipid Chemistry and Nutrition, Oil Crops Research Institute of Chinese Academy of Agricultural Sciences, Wuhan 430062, China; willasa@163.com; 6 Hubei Hongshan Laboratory, Wuhan 430070, China

**Keywords:** *Echium glomeratum*, ω-3 fatty acids, stearidonic acid, pseudotargeted lipidomic profiling, Paternò−Büchi

## Abstract

Currently, ω-3 polyunsaturated fatty acids (PUFAs), which have become popular as dietary supplements, are limited by a shortage in supply. Thus, finding safe, effective alternatives is crucial. *Echium* seed oil (ESO), rich in α-linolenic acid (ALA, 18:3ω-3) and stearidonic acid (SDA, 18:4ω-3), surpasses many other botanical seed oils. In this study, a pseudotargeted approach was applied to characterize the lipidomic profile of two unexplored *Echium* species from the Mediterranean region. Our findings established *Echium glomeratum* as a rich source of ω-3 fatty acids (FAs), exceeding many other species in both quality and quantity. *E. glomeratum* possesses different FAs and triglyceride (TG) profiles compared to *E. judaeum*, with the ω-3:ω-6 ratio being 3.5 and 1.3, respectively. This corresponds to higher quantities of ALA (45.50%) and SDA (12.59%) in *E. glomeratum*. Triglycerides (TGs) comprise 93% of the total lipid content in ESO. This study also profiled the most abundant TGs (50–60 carbons) from both species through comprehensive assignment of the olefination patterns. The *E. glomeratum* oil profile, containing a higher ω-3 PUFA concentration, was further screened for cytotoxic and antioxidant activities. Our preliminary results demonstrated that *E. glomeratum* ESO may significantly suppress colon cancer cell growth.

## 1. Introduction

**Figure 1:**
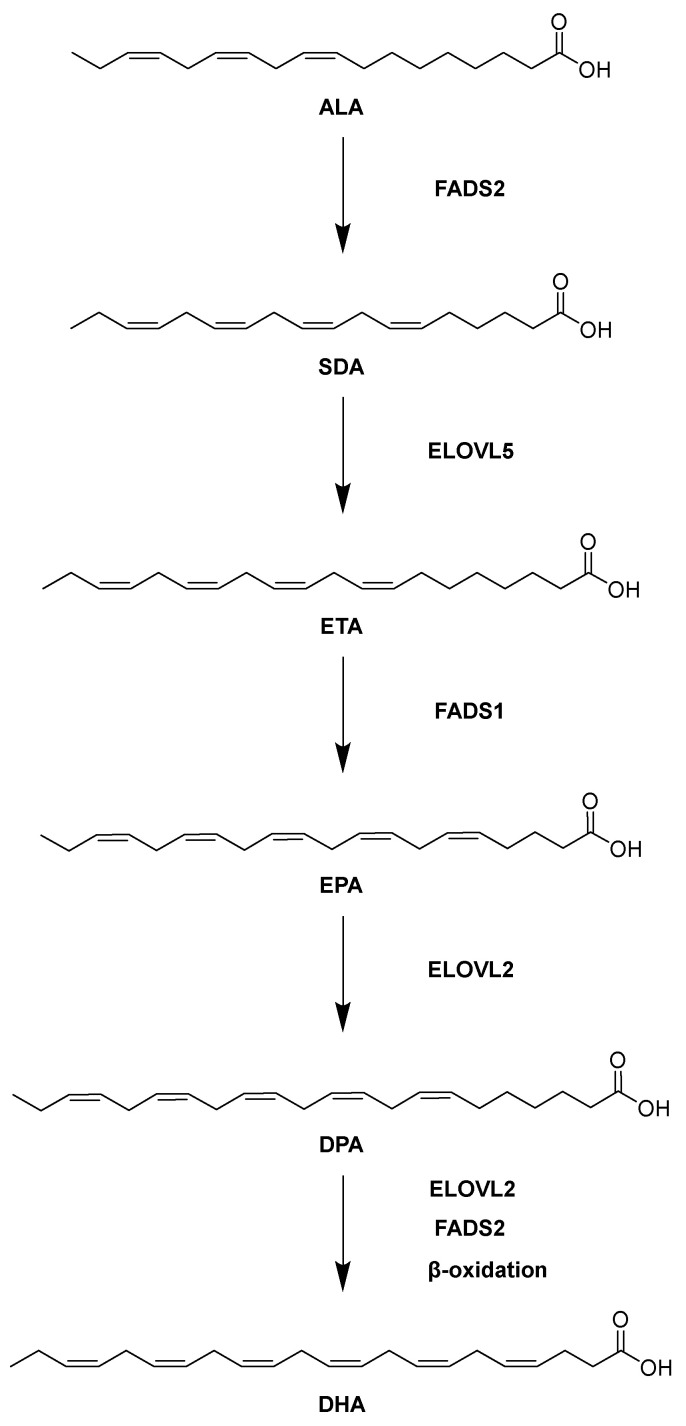
Biosynthetic route to DHA from ALA.

FAs have been a source of contention for the human diet for many years. Currently, dietary FAs are classified as saturated fatty acids (SFAs), monounsaturated fatty acids (MUFAs), polyunsaturated fatty acids (PUFAs), and trans. SFAs garner significant negative attention since they have been demonstrated to raise low-density lipoprotein cholesterol levels in vivo, which is linked to an increase in cardiovascular disease risk. On the other hand, literature has shown that consuming polyunsaturated fatty acids (PUFAs) provides a wealth of health benefits [1]. Of particular importance to human health are the long-chain PUFAs (LcPUFAs), which are involved in immune system regulation, blood clotting, neurotransmission, cholesterol metabolism, and membrane phospholipid structure [2]. Eicosapentaenoic acid (EPA), docosapentaenoic acid (DPA), and docosahexaenoic acid (DHA) are among the most prominent members of the LcPUFAs, primarily due to their varied neuroprotective effects [3]. DHA has been demonstrated to be a critical molecule in the neuronal membrane, while EPA and DPA regulate anti-inflammation [3]. Nevertheless, these key LcPUFAs cannot be synthesized de novo by human metabolic pathways [4]. Instead, literature has identified α-linolenic acid (ALA) as the precursor for EPA, DPA, and DHA anabolism in vivo [4]. As illustrated in Figure 1, ALA is first desaturated by fatty acid desaturase 2 (FADS2) to yield stearidonic acid (SDA). SDA then undergoes chain elongation by elongation of very long-chain fatty acids protein 5 (ELOVL5) to yield eicosatetraenoic acid (ETA). A second desaturation event performed by fatty acid desaturase 1 (FADS1) generates EPA from ETA. EPA is then lengthened by elongation of very long-chain fatty acid protein 2 (ELOVL2) to form DPA. Finally, DPA is converted to DHA by additional elongation, desaturation, and β-oxidation steps. Critically, the activities of the desaturases and elongases involved in LcPUFA synthesis can be influenced by multiple dietary factors, such as abstinence from certain foods, micronutrient availability, and the ratio of LA to ALA in the diet [5]. The health status of the liver also modulates LcPUFA production, since liver disease states such as obesity and non-alcoholic fatty liver disease are related to low LcPUFA levels [6,7]. Moreover, the conversion of ALA to SDA by FADS2 in the first step of the LcPUFA biosynthetic pathway is rate-limiting, yet studies have provided evidence linking elevated expression of FADS2 in tumor cells to increased malignancy [8,9]. Therefore, it is hypothesized that direct supplementation of the diet with SDA or any of the key LcPUFAs (i.e., EPA, DPA, or DHA) would lead to improved LcPUFA availability and would mitigate any negative outcomes of upregulating the expression of certain LcPUFA biosynthetic enzymes.

*Echium*, a genus of more than 60 flowering plants in the Boraginaceae family, has been documented to contain an abundance of PUFAs [10]. Indeed, the ω-3 PUFA composition of ESO is comprised predominantly of ALA and SDA. The relative abundance of SDA in *Echium* is noteworthy, since it is the least abundant FA distributed across the plant kingdom [11]. Consequently, ESO could become a valuable dietary supplement as a source of precursors for LcPUFAs to meet an ever-increasing demand for PUFAs, which currently exceeds supply from aquatic stocks [12,13,14]. Since the ratio of FAs present in plant oils can affect their stability, bioactivity, and absorption, complete characterization of the extracted oil is essential to quality control and formulation efforts [15,16]. To date, only a few *Echium* species have been profiled; therefore, characterization of underexplored species such as *E. glomeratum* and *E. judaeum* could enhance scientific knowledge surrounding the genus [17,18,19,20]. Considering the attractiveness of *Echium* as a natural source of PUFAs, a comprehensive lipidomic characterization of underexplored species was undertaken to identify potential hallmarks of the plant that could optimize *Echium* for PUFA production.

In previous profiling of various plant FA composition, conventional lipid analysis techniques were utilized to roughly estimate the lipid profile of seed oil [21]. For example, the FA composition of seed oils has been determined by gas chromatographic-mass spectrometric (GC-MS) analysis of derivatized fatty acid methyl esters (FAMEs) from crude lipids, which estimates lipid concentration without exact detection of lipid class or double bond location in PUFAs. For oils containing large concentrations of PUFAs, a more comprehensive method for lipid structural characterization and quantification is now afforded by high-resolution mass spectrometry coupled to ultra-high-performance liquid chromatography (HRMS-UPLC) [22]. Recently, an efficient integrated approach known as pseudotargeted analysis was applied, which allowed for a greater detection of lipid classes among analyzed lipid species [16]. In this approach, untargeted (Data Dependent Acquisition (DDA)) lipidome profiling was combined with tandem mass of preselected lipid species to increase the coverage and sensitivity of lipid detection [16,23].

Since this work sought not only to ascertain the lipid classes present in *E. glomeratum* and *E. judaeum* but also to establish the exact FAs isolated in the seed oils, an HRMS-UPLC method was augmented with synthetic approaches. Since the double bonds in PUFAs will not fragment at the regular energy applied with a collision-induced dissociation (CID) system as applied in common MS instruments, the Paternò–Büchi (PB) reaction is carried out on the crude fatty acids obtained from *E. glomeratum* and *E. judaeum*, as previously performed on *Echium plantagineum* seed oil in a previous pseudotargeted lipidomic analysis [16]. Following this, the cytotoxic and the antioxidant activities of *E. glomeratum* oil were assessed by in vitro assays. As a result, this work simultaneously established the crucial nature of the double bond positioning in *E. glomeratum* and *E. judaem* PUFAs, their content was approximately quantified, and their potential cytotoxic effects were screened.

## 2. Results and Discussion

### 2.1. Oil Extraction

The crushed seeds of two *Echium* species were extracted repeatedly with n-hexane using soxhlet apparatus. In this study, samples were collected from multiple harvests and pooled prior to extraction. The aim was to obtain a representative metabolite profile for screening purposes. *E. glomeratum* seed oil was lustrous and yellowish in color, and the yield was 19.4% *w*/*w* g of oil for every 20 g of seeds. Following the same method, the oil content from *E. judaeum* was determined. The oil was transparent and clear, but with a lower yield as compared to *E. glomeratum* (14% *w*/*w* g of oil/20 g of seeds). Previous studies showed ESO content ranges from 7.10 to 23.65% in 19 different species [17]. *E. plantagineum* was shown to have the highest content. The variability in seed oil content is common, and it is affected by multiple factors. Among these factors are the seasonal variability, the source of the seeds, the time of collection, the method of extraction, and storage conditions. In this study, the seeds were collected from the wild, and they reflect two different habitats, especially the soil type where *E. glomeratum* was growing in the red Mediterranean soil, and the *E. judaeum* survives in the xerochrept soil characterized by calcic sub-surface and high content of CaCO_3_. The time of collection of the two species was comparable; however, *E. glomeratum* was collected at the beginning of the season, and *E. judaeum* was collected at the end of the season.

### 2.2. Lipidomic Profile

#### 2.2.1. Fatty Acid Profile

*Echium* is a valuable source of ω-3 PUFA. Hence, we sought to analyze more Mediterranean untapped wild species. Here we have investigated for the first time the PUFA content in *E. glomeratum* and *E. judaeum*, which grow in the Jordan Valley Rift. Our first attempt was the simple analysis of the FA classes by GC-MS. The analysis and quantification of the major FAs by GC-FID are summarized in Table 1. SFAs, MUFAs, and PUFAs were characterized in both species. PUFA can be further classified depending on the location of the first double bond in the chain. Either it will start at carbon 3 (ω-3 PUFA) like ALA, or at carbon 6 (ω-6 PUFA) like Linoleic acid (LA, C18:2n6). Both ALA and LA are essential FAs that should be provided in the diet, and ultimately could be converted into LcPUFAs. However, the consumption of ALA and LA should be in a balanced ratio [24,25]. LA and ALA are common substrates that compete for the enzyme delta-6 desaturase (FADS2) in humans. LA, through the ω-6 pathway, is reported to convert to arachidonic acid (AA, C20:4n6), which in turn is associated with increased production of pro-inflammatory and thrombotic mediators. On the other hand, ALA, the competitor, and through the same pathway, is reported to provide both (EPA, C20:5n3) on average of 10% and (DHA, C22:6n3) on average of 1%, which are associated with anti-inflammatory action [26]. Significant to our study, a notable difference in PUFA profiles was exhibited by the two-representative species of *Echium* growing in the Eastern Mediterranean region in Jordan. However, both showed a high content of the total PUFA, ranging from 67% in *E. judaeum* to 75% in *E. glomeratum* (Table 1). Major PUFAs detected were ALA, LA, SDA, and GLA. Nevertheless, the ω-3 FA content is higher in *E. glomeratum* compared to *E. judaeum* (58% compared to 38%), while *E. judaeum* showed the same trend but with a higher ω-6 content (29% compared to 17%). The ω-3/ω-6 ratio in *E. glomeratum* and *E. judaeum* is 3.5 and 1.3, respectively. This variation stems directly from the difference in LA (C18: 2n ω-6) to ALA (C18: 2n ω-3) in these oils. Previous studies also have shown distinct variability in oil composition and fatty acid content among various *Echium* species [17]. Interestingly, *E. glomeratum* growing in the wild shows higher content of ALA (45.5%) and comparable SDA (12.59%) content as compared to the industrial crops of *E. plantagineum* (ALA 37% and SDA 13%). Multiple studies indicate that these two ω-3 FAs have the potential to convert to LcPUFA in the human body, with SDA being more efficient than ALA. For instance, dietary SDA has shown to increase EPA levels in plasma phospholipids and in red blood cells approximately five times more efficiently than ALA in humans and 35–40% the efficiency of dietary EPA [27]. Collectively, this study found that *E. glomeratum* seed oil is a new wild terrestrial oil rich in SDA, and hence has the potential to decrease inflammation if included in the diet. However, additional studies are needed to validate these effects.

#### 2.2.2. Triglycerides

**Figure 2:**
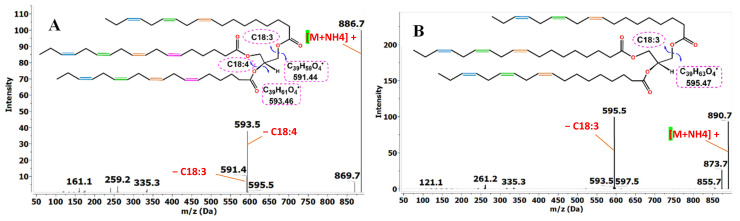
Diagnostic fragments of (**A**) TG54:11 and (**B**) TG54:9 by UPLC-HRMS. (Color code: blue: ω-3, green: ω-6, orange: ω-9, pink: ω-12).

**Figure 3:**
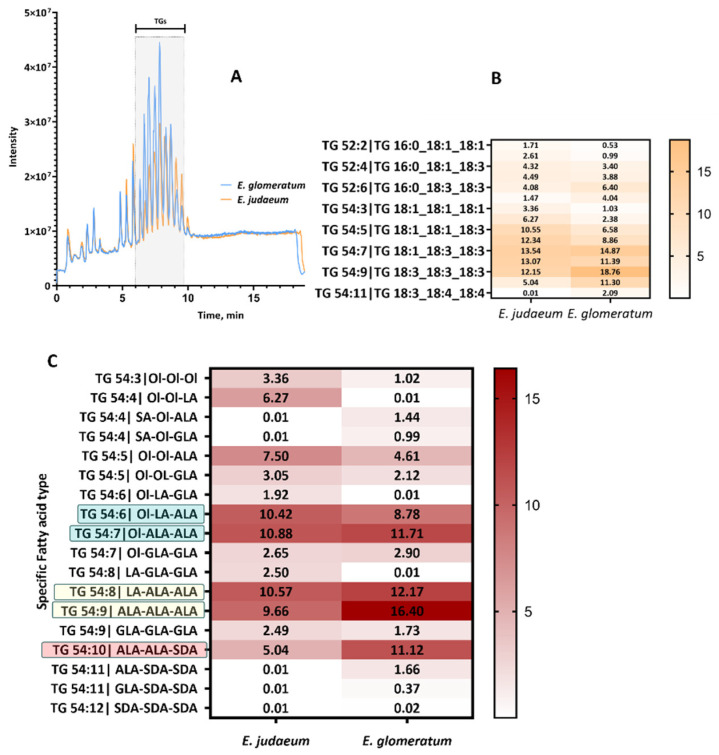
(**A**) Total ion chromatogram by untargeted UPLC-HRMS method (**B**) Most abundant TGs present in ESO in both *E. judaeum* and *E. glomeratum* by the untargeted analysis (**C**) Acyl chains embedded in the most abundant TGs in *E. judaeum* and *E. glomeratum* by the targeted analysis.

The fabric of the TGs present in ESO affects its nutritive value and functional properties. TGs are the most predominant form in ESO, which comprises more than 93% as reported in previous studies [17,28]. Hence, this work was focused on the TG profiles of two wild ESO coming from *E. glomeratum* and *E. judaeum* by integrating tandem mass of preselected lipid species with DDA lipidomics. With the help of the DDA UPLC-HRMS method, the lipid class and the attached acyl chains were defined. Although both ESI+ and ESI− modes were applied, the TG ionizes efficiently and reproducibly in the (ESI+) ion mode, forming ammoniated adducts that are well-suited for MS/MS fragmentation. Since the only analyzed species in this oil is the neutral TGs, the (+)-ion mode was sufficient for the analysis. Briefly, every lipid species is identified according to its retention time, mass accuracy, isotope ratio, and MS/MS fragments [23]. For instance, in the ESI+ source, TG 54:11 fragments distinctively at *m*/*z* 591.4 and 593.5, which can be translated directly to the loss of the main fatty acyl chains C18:3 and C18:4 (Figure 2A). Similarly, TG 54:9 (C18:3_C18:3_C18:3) has an intense peak at *m*/*z* 595.4724, indicating the loss of C18:3 as the major fragment (Figure 2B). In this study, the TGs in the range of 50–60 carbon atoms (total carbons in all acyl chains) were elucidated and approximately quantified in both *E. glomeratum* and *E. judaeum*. Although no internal standard normalization was attempted in this study, the main TGs were approximately quantified and were presented as the average relative content in mg/100 mg ± SD of two replicates of the same sample, as shown in Table S1. The relative content of each TG was calculated with the assumption of the high-purity glyceride extracted with hexane. This semi-quantification method is based on the principle that the molar concentration ratio of the compound to the internal standard equals the ratio of their peak areas. The 50–60 specified range encompasses the most abundant TGs, specifically those rich in the ω-3 and ω-6 FAs. By the application of this method, more than 50 TGs were identified and approximately quantified in both species (Figure 3A). These 50 TGs comprise more than 90% of the oil and 25% of the total number of species that could be detected by this method. Although we have focused only on TG with this specific range, a more comprehensive lipidomic profile could be revealed, including TG range from 30 to 50 carbons, diacylglycerides, sterol esters, and free FAs. Collectively, these unidentified species represent less than 8% of the oil content relative to the total TIC area. More comprehensive *Echium* oil lipidomic analysis and alternative fragmentation patterns are discussed in detail in the previous work of Xu et al. 2021 [16], which was focused on analyzing the commercial oil *E. plantagineum*. The results of our DDA UPLC-HRMS analysis and approximate estimation are in accordance with the GC analysis, in which the latter method is known to be highly accurate and reproducible. In both *Echium species*, the TG 54:5–10 species represented the most abundant triglycerides and exhibited the highest C18:3 content, with some degree of variation as shown in Figure 3B. In *E. judaeum*, TG 54:5 (C18:1_C18:1_C18:3), TG 54:6 (C18:1_C18:2_C18:3), TG 54:7 (C18:2_C18:2_C18:3 and C18:1_C18:3_C18:3), TG 54:8 (C18:2_C18:3_C18:3), TG 54:9 (C18:3_C18:3_C18:3) were the most abundant TGs comprising 10.83 ± 0.40%, 12.25 ± 0.13%, 13.42 ± 0.16%, 12.16 ± 1.29, and 11.06 ± 1.54%, respectively. TG 54:8 and TG 54:9 were the richest in C18:3. In *E. glomeratum* the most representative species were TG 54:6 (C18:1_C18:2_C18:3), TG 54:7 (C18:1_C18:3_C18:3), TG 54:8 (C18:1_C18:3_C18:4/ and C18:2_C18:3_C18:3), TG 54:9 (C18:3_C18:3_C18:3), and TG 54:10 (18:3_18:3_18:4) of 9.09 ± 0.36%, 14.88 ± 0.85%, 11.39 ± 2.78, 17.37 ± 0.58, and 9.89 ± 0.44%, respectively. The most characteristic TG in *E. glomeratum* is TG 54:9, comprising 17.37% of the total TG, which has three C18:3 acyl chains. In contrast to other plant seed oils, ESO is a valuable source of SDA. SDA is distinguished by its potential conversion to LcPUFAs, and it is correlated with many health benefits as reported in previous studies [29]. As a result, it is trending now to add those to the processed food and skin care products. Interestingly, *E. glomeratum* has approximately considerable amounts of SDA C18:4 compared to *E. judaeum*, and comparable to the commercial oil *E. plantagineum*, which was detected in TG 52:7, TG 54:8, TG 54:10, TG 54:11, and TG 54:12. Regarding our analysis methods, some universal and widespread phenomena such as co-elution, carry over and ion suppression are inevitable; however; the chromatographic conditions were optimized to reduce them. For instance, a very low concentration of the injected sample is applied. At higher concentrations, an ion-suppression effect among TGs could happen, and compounds may undergo aggregation, forming dimers and trimers. Reducing the concentration of the injected sample avoids these effects as much as possible. Also, since this suppression effect applies to all triglycerides, it does not affect our ability to identify the triglyceride species with the highest content. Regarding co-elution, the high mass accuracy threshold of <3 ppm and isotopic pattern matching help in differentiating co-eluting species. For example, TG isomers (16:0/18:1/18:0) versus (16:0/18:0/18:1) can be distinguished by their fragmentation patterns (characteristic DAG fragments at *m*/*z* [M-NL]^+^). In conclusion, the utilized DDA UPLC-HRMS method provided us with the major TG classes, identified the attached acyl chain classes, and approximately quantified these TG species. Next, further examination of the C18:3 isomers present in these triglycerides has been done to determine the exact acyl chain identity in each TG.

#### 2.2.3. Localization of Lipid Double Bonds and Discrimination of Isomers in Echium Oil

**Figure 4:**
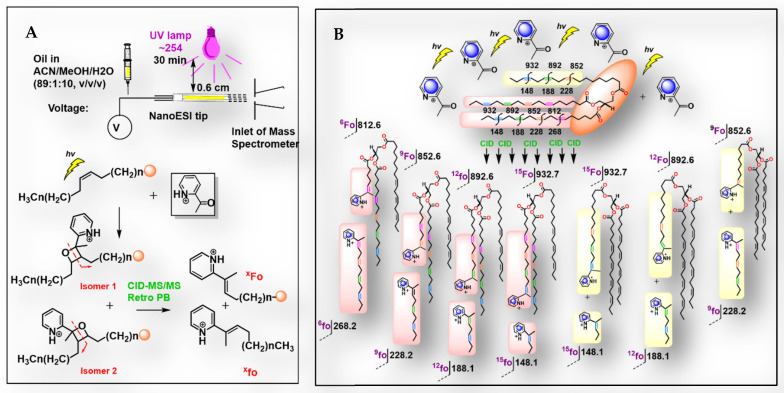
(**A**) Paternò–Büchi derivatization reaction for the unsaturated fatty acids by 2-AP reagent and the resulting major products (**B**) TG54: 11 major retro-fragments. Two types of fragments are represented: a larger fragment is the one connected to the rest of the acyl glyceride (designated here as xFo, where x indicates the position of the double bond according to the Δ nomenclature), and a smaller free fragment (designated as xfo).

Untargeted analysis of the oil enabled the identification of lipid classes and acyl chain types; however, it could not reliably distinguish specific PUFA isomers in TGs. Therefore, a targeted approach with photochemical derivatization was applied to determine double bond locations. Previous studies demonstrated the applicability of the PB reaction in positioning of the double bonds, by utilizing 2-acetylpyridine (2-AP) as the derivatizing reagent [16]. Coupling photochemistry with tandem mass spectrometry offers a direct insight into the double bond locations in PUFA with a high sensitivity and reproducibility (Figure 3C). This technique tags the double bonds by PB reaction and converts them into an oxetane ring by [2+2] cycloaddition, creating two regioisomers (Figure 4A). The oxetane ring fragments at lower-energy collision-induced dissociation (CID), producing distinctive fragments for the two regioisomers. The validation of the method with a defined standard mix of TG 54:3 18:1(9Z)/18:1(9Z)/18:1(9Z), and TG 54:9 18:3(9Z,12Z,15Z)/18:3(9Z,12Z,15Z)/18:3(9Z,12Z,15Z) was carried out previously [16]. Briefly, under CID conditions, these 2-AP-labeled standards have generated characteristic fragment ions (*m*/*z* 1006.9 and 994.8, respectively) with a 122 Da difference compared to the unlabeled species (*m*/*z* 902.8 and 890.7). For clarification, the following detailed example is provided. Labeling TG 54:11 (C18:3_18:4_18:4) (*m*/*z* 868.6) by PB reaction was observed by detecting a sharp peak at *m*/*z* 990.8 (+122). Next, activation by collisions in the CID mode has generated distinct fragments pairs (Figure 4: xfo/xFo) at *m*/*z* 148.1/932.7, 188.1/892.6, 228.2/852.6, 268.2/812.6, which facilitates its identification as shown in detail in Figure 4B. The length of the fatty acyl chains in the labeled TG 54:11 can be identified by the peaks at *m*/*z* 591.4 and 593.5 that match the neutral loss of C18:3 and C18:4, respectively. The presence of the pair peak 268.2/812.6 indicates the oxetane ring formation at the Δ6 double bond and the formation of 2-AP-labeled fragments. Similarly, *m*/*z* 228.2/852.6 is concluded as Δ9, 188.1/892.6 as Δ12, and 148.1/932.7 as Δ15. Collectively, TG54:11 could be assigned to the structure TG 54:11 18:3(Δ9, Δ12, Δ15)_18:4(Δ 6, Δ9, Δ12, Δ15)_18:4(Δ6, Δ9, Δ12, Δ15). Although other fragmentation paths are possible [16], the presented fragmentation is the prevalent one. The acyl chains embedded in 50 TGs were determined via the pseudotargeted approach in two *Echium* species. The results of this analysis are presented as the average relative content (mg/100 mg) ± SD of two replicates of the same sample in Table S2. The assignment of the representative TG found in ESO in both species can be found in Table 2. Although more than one hundred TG species could be detected in this method, only a few are characteristic for each ESO (TGs 54:5–10: Figure 3C). Data analysis of the ESO of *E. glomeratum* and *E. judaeum* revealed major differences in the C18:3 fatty acyl chains’ specific identity. The predominant TG in *E. glomeratum* is TG 54:9 with two variants. The first one has three ALA acyl chains (AlA-AlA-AlA) and comprises the highest percentage (17.90 ± 2.122), and the second one has three GLA acyl chains (GLA-GLA-GLA) and shows only 1.34 ± 0.551%. On the other hand, in *E. judaeum*, TG 54:9 showed less content of the ω-3 variant (AlA-AlA-AlA: 8.86 ± 1.130) and higher content of the ω-6 variant (GLA-GLA-GLA: 2.27 ± 0.320). Indeed, this difference reflects our GC analysis results (2.2.1). In *E. glomeratum*, ALA is also found in TG 54:6, 7, 8, 10 in good amounts. This corresponds with the higher ω-3/ω-6 ratio observed in this species. SDA was detected mainly in TG 54:10. This result is consistent with previously reported findings identifying the major TG in *E. plantagineum* [16]. Even though SDA was identified in both species in TG 54:10 and TG 54:11, *E. glomeratum* has double the amount as compared to the *judaeum* species. The relative abundance and the identity of TG 54:10 and TG 54:11 in these two species are as follows: 11.30 ± 0.254% (ALA-ALA-SDA), 1.91 ± 0.360% (ALA-SDA-SDA) in *E. glomeratum,* as compared to 4.54 ± 0.710% (ALA-ALA-SDA) and 0.59 ± 0.010 (ALA-SDA-SDA) in the *judaeum* species. Furthermore, SDA (C18:4) was part of TG 54:8 in *E. glomeratum* but not in *E. judaeum* (Table 2). The characterization of high levels of ALA and SDA in *E. glomeratum* oil, and their specific distribution within the TG, emphasizes its potential as a valuable functional food ingredient. Owing to its more distinctive TG profile and higher ω-3 FAs content, we have run preliminary studies exploring its potential biological activities.

### 2.3. In Vitro Biological Evaluation of the E. glomeratum Oil

The antiproliferative effect of *E. glomeratum* oil was assessed in vitro against the human colorectal cancer cell line HCT116. The results are reported as IC_50_ values, representing the concentration required to inhibit 50% of cell viability. The IC_50_ of the *E. glomeratum* oil against the examined cell line was 0.08 ± 0.01 (*v*/*v*) after 24 h treatment compared to 5 µM for doxorubicin (positive control). The oil did not exhibit any potential cytotoxicity against normal dermal fibroblasts in the examined range of concentration (0.01–1) after 24 h treatment, suggesting a reasonable safety profile of the oil. Previous studies have shown the cytotoxic effect of PUFA against multiple cell lines [30,31]. However, this assay is only done for screening, and more comprehensive studies should be done to verify the role of ω-3 FAs in this cytotoxic activity, for instance, including representative fatty acids found in the extract in the test, and the control of lipid oxidation products, which might interfere with the results. Since it is well-known that the food items or oils containing PUFA are more prone to peroxidation, they are also prone to deterioration. To assess the quality of the total antioxidant activity of the methanolic fraction of *E. glomeratum* oil, the radical scavenging activity was assayed. The trolox equivalent antioxidant capacity (TEAC) was 0.70 mg (trolox mg/mL to mg of oil), which shows that the oil has half of the antioxidant capacity as compared with trolox, an analogue to vitamin E. This indicates a moderate radical scavenging activity. As reported in earlier studies, the antioxidant activity of ESO is mainly due to the presence of γ-tocopherol and other polyphenols [32]. However, more studies are required to verify the identity of compounds responsible for the antioxidant activity.

## 3. Materials and Methods

### 3.1. Chemicals and Reagents

Acetonitrile (ACN), methanol (MeOH), isopropyl alcohol (IPA), ammonium acetate, and chloroform (CHCl_3_) were supplied by CNW (Düsseldorf, Germany), and they were all HPLC−MS-grade. Ultrapure water was prepared by a Milli-Q system (Millipore, Bedford, MA, USA). 2-acetylpyridine (2-AP) was obtained from Aladdin (Shanghai, China). TG standards, including TG 18:1(9Z)/18:1(9Z)/18:1(9Z), TG 18:3(9Z,12Z,15Z)/18:3(9Z,12Z,15Z)/18:3(9Z,12Z,15Z), were purchased from Larodan Fine Chemicals (Malmo, Sweden). DPPH and TROLOX were obtained from Sigma Aldrich (St. Louis, MO, USA). Dulbecco’s Modified Eagle’s Medium (DMEM) supplemented with 100× L-glutamine, and 100× penicillin/streptomycin was supplied by EuroClone, (Milan, Italy).

### 3.2. Echium Seed Collection

The mature seeds of two species from the *Echium* genus (*E. glomeratum* and *E. judaeum*) were collected from their natural habitat in Jordan. Both species were identified by Dr. Mohammad Alghraibeh, and voucher specimens were prepared and kept at the Faculty of Pharmacy, Philadelphia University. *E. glomeratum* species, a tall biennial or perennial herb, 60–200 cm with an erect, stout stem branched from the base and ending in a long, narrow inflorescence, was collected from Aydoun province, a low scrubland ecoregion (Irbid, Jordan, 32°52′61.7″ N latitude, 35°85′29.6″ E longitude, and 1495 m altitude) in early June 2021. The soil in this region is the red Mediterranean soil, and the average low to high temperatures range between 18 and 30 °C. While *E. judaeum* Lacaita, a shorter annual herb, 25–50 cm with a branching stem from above, was collected from the Philadelphia University campus (Amman, Jordan, 32°09′54.1″ N 35°51′04.6″ E, and 1000 m altitude) in July 2021. The soil in this mountainous region is classified as xerochrept, characterized by calcic sub-surface and high content of calcium carbonate. The average low to high temperatures are between 22 and 32 °C in July. This study is focused on technical replicates of pooled biological replicates from multiple harvests from the same location and during the same season. We followed this strategy to generate a robust average chemical profile suitable for our initial preliminary screening. However, variability among individual plants and across different harvests was not systematically assessed. Plants were included based on species identity, comparable developmental stage, and excluded for any visible disease or damage. The seeds were collected from fully ripened pods, cleaned carefully, and kept in a cold and dark area for further use. Only ripened, fully matured seeds were included.

### 3.3. Echium Oil Extraction

The seeds (20 g) were sampled from the pooled seed harvest for each species. Then they were crushed by mortar and pestle, and then extracted with n-hexane (400 mL) by Soxhlet extractor in 12 cycles at 50 °C. The collected extract was stirred with magnesium sulfate and filtered. Then, the solvent was removed by a rotatory evaporator at 40 °C. After that, the oil was collected, flushed with nitrogen gas, and weighed accurately before it was kept at −80 °C for further use.

### 3.4. PUFA and TGs Profiling

#### 3.4.1. GC-MS Analysis for Verification of the PUFA Types

The methyl ester derivatives of the fatty acids known as (FAMEs) were prepared as previously described [33]. The internal standard was methyl heptadecanoate (C17:0). The derived esters were analyzed by an Agilent 7890A gas chromatograph (GC) coupled with a flame ionization detector (FID) (Agilent, Palo Alto, CA, USA). The installed capillary column was DB-FastFAME (30 m × 0.30 mm, 0.25 μm, Agilent J&W, Folsom, CA, USA), the carrier gas was nitrogen (purity ≥ 99.999%); the temperature of injection was 250 °C, injection volume, 2 μL; split ratio, 1:20; the temperature of column oven increased from 80 to 165 °C at 40 °C/min (held for 1 min), and then increased from 165 to 230 °C at 4 °C/min (held for 4 min), the detector temperature was set to 260 °C. All experiments were carried out in duplicates, and the data are presented as the mean ± SD.

#### 3.4.2. Preparation of Samples

To prepare the samples, 10 mg of ESO of two species were solubilized with 10 mL of CHCl_3_/MeOH (2:1, *v*/*v*) and vortexed for 1 min. Then, it was diluted to 30 µg/mL using the same solvent system, and the prepared samples were stored at −20 °C till further analysis.

#### 3.4.3. UPLC-HRMS Conditions for DDA Lipidomics

A Shimadzu 30A UPLC system (Kyoto, Japan) hyphenated with a Triple TOF 6600 mass spectrometer (Q-TOF MS, Sciex, Toronto, ON, Canada) was used for DDA acquisition. Diluted ESO aliquots (10 µL) were injected into a C18 column (100 × 2.1 mm, 2.6 μm, Phenomenex Kinetex, Torrance, CA, USA). The mobile phase is composed of two parts. Part A is MeOH/ACN/water (1:1:1, *v*/*v*/*v*) and part B is IPA/ACN (5:1, *v*/*v*), both with 5 mM ammonium acetate added. The UPLC pump was programmed at a 0.4 mL/min flow rate. The optimal gradient program was applied as follows: 20% B at 0–0.5 min, 20–40% B at 0.5–1.5 min, 40–60% B at 1.5–3 min, 60–95% B at 3–13 min, 95% B at 13–18 min, 95–20% B at 18–18.1 min, and 20% B at 18.1–20 min. DDA was performed for data acquisition, and both electrospray ionization (ESI+ and ESI−) were performed. MS parameters were as follows: the declustering potential (DP) was set to 80 V, the collision energy (CE) and the ion spray voltage were set to 30 V and 5500 V, respectively, with a mass range from *m*/*z* 50 to 1200. Gas1 and gas 2 were both set to 50 psi. Curtain gas was set to 35 psi, and the interface heater temperature was at 600 °C. All experiments were carried out in duplicates, and the data are presented as the mean ± SD of the relative contents. The mass accuracy is <3 ppm with external TOF calibration run every 5 samples using standard calibration solution. The theoretical dynamic range is 4–5 orders of magnitude. The Scan Speed was 100 Hz for MS/MS, and the resolving Power was ~30,000 FWHM (TOF MS mode) and ~15,000–20,000 FWHM in high-sensitivity MS/MS mode.

#### 3.4.4. Derivatization of Unsaturated TGs with PB Reagent

A reaction mixture of 1–2 mM of 2-AP with TG standard (1–10 µg/mL) or ESO samples (200 µg/mL) was prepared in ACN/MeOH/H2O (89:1:10, *v*/*v*/*v*) containing ammonium acetate (20 mM). To carry out the PB reaction, the reaction mixture was transferred to quartz cuvettes (1.4 mL, 10 mm light path, Yixing Chenwei Glass Instrument factory, Yixing, China). The cuvette was positioned 0.6 cm from the low-pressure mercury lamp (254 nm, model 80-1057-01, BHK, Inc., Claremont, CA, USA). A 30 min ultraviolet (UV) exposure of the reaction mixture was followed. All experiments were carried out in duplicates, and the data are presented as the mean ± SD.

#### 3.4.5. Conditions for Shotgun MS to Specify TG Double Bonds

The assignment of double bonds was made after UV irradiation. The product analysis of the PB reaction was performed by an ESI-MS/MS system composed of a Triple TOF 6600 MS spectrometer (AB Sciex, Toronto, ON, Canada) equipped with a syringe pump. Relative semi-quantitative analysis of derivatives of unsaturated lipids was performed in (+) ion mode. The flow rate of derivatives infused via the syringe pump was 5 µL/min, and the syringe volume was 1 mL. The parameters of the instrument were optimized to achieve the highest [M+H]+ ion abundance by infusing derivatives via a syringe pump into the MS with an optimal flow of 5 μL/min. The pressure of the collision gas was high; the curtain gas pressure and the nebulizer pressure were 30.0 and 45 kPa, respectively. The temperature was 550 °C. The ion source gas 1 and ion source gas 2 were assigned to 25 psi, and the DP to 100 V. The mass range was set to *m*/*z* 50–1100. CE: 55–65 V.

#### 3.4.6. DDA Data Processing

DDA lipidomics datasets generated using a UPLC–TripleTOF 6600 mass spectrometer were processed using the open-source software MS-DIAL (v4.24; http://prime.psc.riken.jp/compms/msdial/main.html accessed on 17 August 2023) as well as proprietary tools including PeakView (v2.2), MasterView (v2.0), and MultiQuant (v3.0.3) (AB Sciex, Toronto, Canada). Initial lipid annotation was performed with MS-DIAL following conversion of the raw instrument files (.wiff) into Analysis Base File (.abf) format. Metabolite identification was based on a combination of chromatographic retention behavior, accurate mass measurements, isotopic pattern evaluation, and tandem mass spectrometry spectral matching against the LipidBlast reference libraries.

### 3.5. Cell Viability Assay

3-(4,5-dimethylthiazol-2-yl)-2,5-diphenyltetrazoliumbromide (MTT) assay was used to evaluate cell proliferation as previously noted [34]. Human CRC cell line (HCT116) and normal dermal fibroblast cells were seeded in 96-well plates at variable seeding density per well, depending on doubling time, proliferation ability, and target time of treatment. Cells in wells were seeded in DMEM medium and incubated at 37 °C in a humidified incubator set at 95% humidity and 5% CO_2_. After 24 h, the media were discarded to enable accurate treatment concentration as desired. Gradient micromolar concentration points (*v*/*v*) were applied for both the oil sample and the positive control (doxorubicin). In addition, wells containing fresh DMEM were used as negative controls, which were prepared under the same conditions. After treatment time elapsed, 10 µL of MTT dye at a working concentration of 5 mg/mL was added to each well, followed by plate incubation for 3 h in the dark at 37 °C in a humidified incubator set at 95% humidity and 5% CO_2_. Once the incubation period elapsed, the media was gently aspirated using a 1 mL clean insulin syringe so as not to disturb the formed formazan crystals. Once the media is aspirated, DMSO 100 µL was added to each well to solubilize the crystals, and plates were put on an orbital shaker at 100 RPM for 15 min in a dark environment. Optical density was measured at 570 nm and 630 nm using a microplate reader (µ Quant Plate Reader, Biotek, Winooski, VT, USA). All experiments were run in triplicate wells and repeated at least two times independently. The percentage of the relative cell viability of treated cells versus the untreated cells (negative controls) was calculated using the following formula in Equation (1).

(1) $$Cell\;viability\left(\%\right)=\frac{Optical\;density\;of\;treated\;cells}{optical\;density\;of\;untreated\;cells}\times 100$$

### 3.6. Antioxidant Activity

The radical scavenging activity (RSA) of E. glomeratum oil was evaluated against the 1,1-diphenyl-2-picryl-hydrazyl (DPPH) radical. The oil was extracted with methyl alcohol by continuous shaking of 100 mg of the oil in 5 mL of methanol for 15 min. After decantation, the upper methanolic layer was taken, and multiple dilutions were made to calculate RSA. The antioxidant activity of the extract and standards was measured according to the method published by Atolani et al. [35]. In brief, 100 µL of 0.1 mM of methanolic DPPH was mixed with either 100 µL of the test samples with varying concentration (15, 7.5, 3.75, 1.5, 0.75 mg/mL) or 100 µL of trolox standard at varying concentrations (12.5, 10, 6.25, 5, 2.5, 1.25 mg/mL). The mixture was incubated for 30 min in the dark. The absorbance of the samples and control was measured at 517 nm using a microplate reader (multiskan, Thermo Scientific, Waltham, MA, USA) using flat-bottom 96-well polystyrene microplates (300 µL/well). The RSA was expressed as trolox equivalent antioxidant capacity (TEAC, mg/mL trolox equivalents per mg of oil).

## 4. Conclusions

ESO is known for its unique FA composition and health benefits. According to dietitians, this oil stands for its balanced ω-3/ω-6 ratio and the high SDA content, which is rarely observed in other plant seed oils. In the current study, and by the application of pseudotargeted lipidomics, we have profiled the most abundant TGs in two underexplored *Echium* species from the Mediterranean region: *E. glomeratum* and *E. judaeum*. The additional positioning of double bonds in these FAs was accessed via PB reaction with 2-AP derivatization. Accordingly, the most abundant TGs (50–60 carbons) were profiled in both species. In terms of ω-3 FAs content, *E. glomeratum* showed a better profile as compared to *E. judaeum*. The oil content of the seeds was determined to be close to 20%. ALA and SDA (45.50 ± 0.022% and 12.59 ± 0.063%) were the dominant species. TG 54:9 was the most representative in this species with 19.24% relative content, which breaks down into 17.90 ± 2.122% of ALA-ALA-ALA structure, and 1.34 ± 0.551% of GLA-GLA-GLA. SDA, the distinctive LcPUFA-direct precursor, was observed mainly in TG 54:10 (11.30 ± 0.254% as ALA-ALA-SDA), TG 54:11 (1.91 ± 0.360% as ALA-SDA-SDA), and to a lesser amount in TG 54:12 (0.03 ± 0.003% as SDA-SDA-SDA). This study is a preliminary evaluation of the specific TG content, and it is not a comprehensive quantitative study. A complete quantitation workflow of the ω-3 FAs isomers found in the oils of the wild Boraginaceae seeds will be conducted in the future. Owing to the interesting FAs and TGs composition of *E. glomeratum*, we have assessed its cytotoxicity and antioxidant effects in vitro. This oil exhibited significant inhibitory effects on colon cancer cell growth and presented moderate antioxidant activity, which might further add to its nutritive value. Further in vitro and in vivo studies are needed to verify these roles. In summary, this study explored a new plant resource for PUFA, including ALA and SDA. The PB reaction applied here, coupled to UPLC-HRMS, expedites the analysis of TG composition and the double bond positioning. Future refinements for the analytical method will be directed toward automation and precise determination of fatty acid positions within the corresponding TG.

## Figures and Tables

**Table 1:** FA composition in the oil extracted from *E. judaeum* and *E. glomeratum*. The results of the analysis and quantification (GC-FID) are presented as %, *w*/*w* means ± SD.

FA Type	FA Name	FA Abbreviation	*E. judaeum*	*E. glomeratum*
C16:0	Palmitic acid		7.21 *±* 0.002	6.91 *±* 0.001
C16:1n-7	Palmitoleic acid		0.06 *±* 0.005	0.06 *±* 0.009
C18:0	Stearic acid		3.78 *±* 0.001	2.94 *±* 0.004
C18:1n-9	Oleic acid	Ol	21.04 *±* 0.080	14.74 *±* 0.040
C18:2n-6	Linoleic acid	LA	21.95 *±* 0.060	11.06 *±* 0.048
C18:3n-6	γ-linolenic acid	GLA	6.93 *±* 0.004	5.065 *±* 0.009
C18:3n-3	α-linolenic acid	ALA	31.77 *±* 0.015	45.50 *±* 0.022
C18:4n-3	Stearidonic acid	SDA	6.36 *±* 0.009	12.59 *±* 0.063
C20:1n-9	Eicosenoic acid		0.67 *±* 0.005	0.51 *±* 0.007
C20:2n-6	Eicosadienoic acid		0.24 *±* 0.004	0.03 *±* 0.004
∑SFA			10.98	9.85
∑MUFA			21.78	15.29
∑PUFA			67.24	74.83
∑ω-3			38.13	58.09
∑ω-6			29.12	16.74
∑ω-9			21.71	15.26
ω-3/ω-6			1.3	3.5

**Table 2:** Characterization of the FAs isomers in the prominent triglycerides range (TG 54:1-12) in *E. judaeum* and *E. glomeratum* by the targeted approach (data are represented as relative content of TG means (mg/100 mg) ± SD). The characteristic 2-AP-labeled parent ion (*m*/*z*) for each TG is also presented.

TG	Observed Combinations	*^a^*% *E. judaeum*	*^a^*% *E. glomeratum*	Double Bond Location in Unsaturated Fatty Acids	2-AP-Labeled Parent Ion (*m*/*z*)
TG54:1	SA-SA-OL	0.12 ± 0.055	0.04 ± 0.017	18:1(△9)	1010.9
TG54:2	SA-OL-OL	1.16 ± 0.035	0.23 ± 0.012	18:1(△9)	1008.9
TG54:3	OL-OL-OL	4.03 ± 0.960	1.03 ± 0.011	18:1(△9)	1006.9
TG54:4	OL-OL-LA SA-OL-ALA SA-OL-GLA	6.81 ± 0.760 ND ND	ND 1.41 ± 0.043 0.97 ± 0.025	18:1(△9) 18:2(△9,△12) 18:3(△9,△12,△15) 18:3(△6,△9,△12)	1004.8
TG54:5	OL-OL-ALA OL-OL-GLA	8.05 ± 0.780 2.81 ± 0.330	4.49 ± 0.173 2.09 ± 0.049	18:1(△9) 18:3(△9,△12,△15) 18:3(△6,△9,△12)	1002.8896
TG54:6	OL-LA-ALA OL-LA-GLA	9.75 ± 0.950 2.36 ± 0.620	8.93 ± 0.200 ND	18:1(△9) 18:2(△9,△12) 18:3(△9,△12,△15) 18:3(△6,△9,△12)	1000.8725
TG54:7	OL-ALA-ALA OL-GLA-GLA	9.80 ± 1.540 3.51 ± 1.200	11.82 ± 0.158 3.040 ± 0.198	18:1(△9) 18:3(△9,△12,△15) 18:3(△6,△9,△12)	998.8579
TG54:8	(OL-ALA-SDA/ LA-ALA-ALA) (OL-GLA-SDA/ LA-ALA-ALA) LA-ALA-ALA LA-GLA-GLA	ND ND 10.00 ± 0.828 2.32 ± 0.264	10.79 ± 1.943 1.10 ± 0.126 ND ND	18:2(△9,△12) 18:3(△9,△12,△15) 18:3(△6,△9,△12) 18:4(△6,△9,△12,△15)	996.8426
TG54:9	ALA-ALA-ALA GLA-GLA-GLA	8.86 ± 1.132 2.27 ± 0.319	17.90 ± 2.122 1.34 ± 0.551	18:3(△9,△12,△15) 18:3(△6,△9,△12)	994.7271
TG54:10	ALA-ALA-SDA	4.54 ± 0.708	11.30 ± 0.254	18:3(△9,△12,△15) 18:4(△6,△9,△12,△15)	992.8128
TG54:11	ALA-SDA-SDA GLA-SDA-SDA	0.59 ± 0.012 ND	1.91 ± 0.360 0.48 ± 0.167	18:3(△9,△12,△15) 18:3(△6,△9,△12) 18:4(△6,△9,△12,△15)	990.7979
TG54:12	SDA-SDA-SDA	ND	0.03 ± 0.0003	18:4(△6,△9,△12,△15)	988.775

*^a^* Percentages are expressed as mg/100 mg.

## Data Availability

The original contributions presented in this study are included in the article/Supplementary Material. Further inquiries can be directed to the corresponding author.

## Supplementary Materials

The following supporting information can be downloaded at: https://www.mdpi.com/article/10.3390/molecules31030550/s1, Table S1: Untargeted TG identification; Table S2: Untargeted TG identification_Mass errors; Table S3: Pseudotargeted lipidomics.

## Abbreviations

The following abbreviations are used in this manuscript:

AA	Arachidonic Acid
2-AP	2-acetylpyridine
ALA	α-linolenic acid
CID	Collision-Induced Dissociation
DHA	Docosahexaenoic acid
DDA	Data Dependent Acquisition
ELOVL5	Elongation of very long-chain fatty acids protein 5
EPA	Eicosapentaenoic acid
ESO	*Echium* seed oil
ETA	Eicosatetraenoic acid
FADS1	Fatty acid desaturase 1
FADS2	Fatty acid desaturase 2
FAMEs	Fatty acid methyl esters
GC-MS	Gas chromatography coupled to mass spectrometry
LcPUFA	Long-chain PUFA
MUFAs	Monounsaturated fatty acids
PUFA	Polyunsaturated fatty acids
SDA	Stearidonic acid
TG	Triglycerides
SFAs	Saturated fatty acids
UPLC-HRMS	Ultra-high-performance liquid chromatography, high-resolution MS
